# Supplementary material for: Transcriptome and Metabolome Integrated Analysis of Two Ecotypes of Tetrastigma hemsleyanum Reveals Candidate Genes Involved in Chlorogenic Acid Accumulation
Source: Plants (Basel). 2021 Jun 24;10(7):1288. doi: 10.3390/plants10071288 (PMC8309080; doi:10.3390/plants10071288)
Supplement: Supplementary file 1 [file plants-10-01288-s001.zip › plants-1247736-supplementary.pdf]

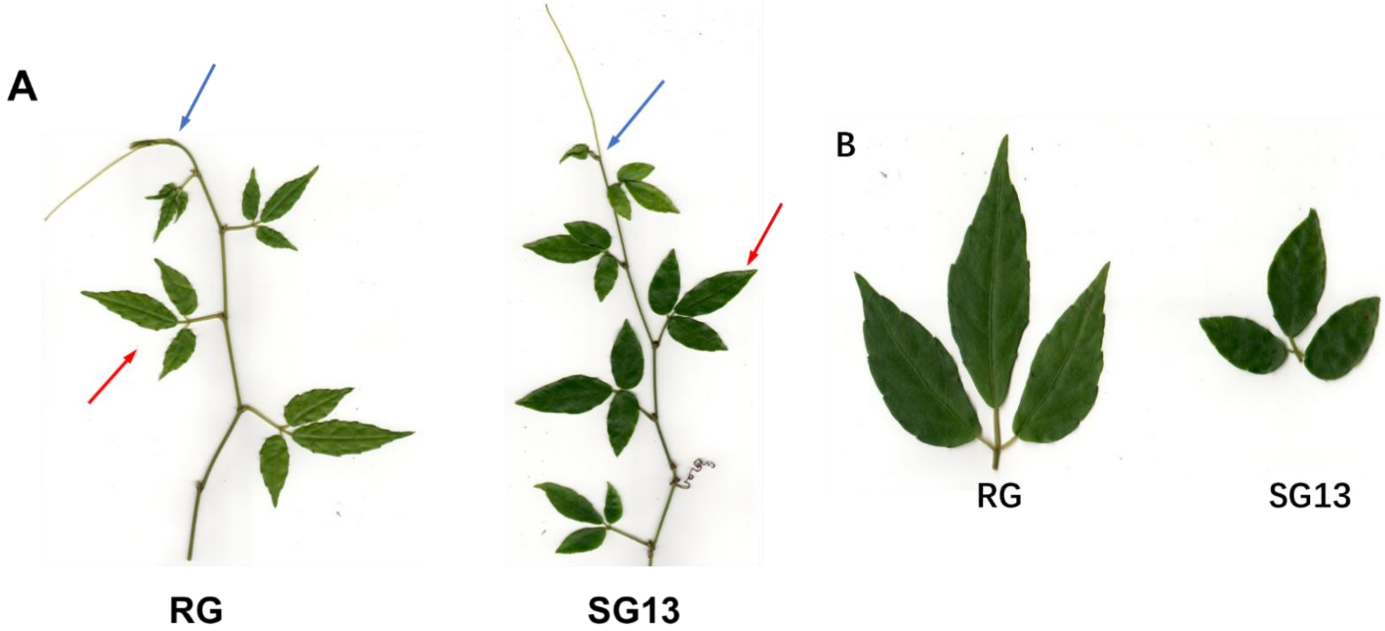

Figure. S1 Phenotypes of medium-sized-leaf SYQ and small SYQ.

(A) Red arrows indicate the leaves collected for analysis; blue arrows indicate the top of stem.

(B) Exhibited representative leaves of RG and SG13.

Towards plant salinity tolerance-implications from ion transporters and biochemical regulation

Table S1. Information of genes and primers for qRT-PCR

Accession number	Function prediction	Primer Sequences(5' to 3')
	SYQ EF1α	CGGGCAAGTCCACCACTACT
		CATCTCAGCAGCCTCCTTCTCAAA
c102511.graph_c0	SYQ CCoAOMT	CAAGAGACTGATCGATTTGGTGAAG
		GTTCCAGAGGGTGTTGTCGTA
c94054.graph_c0	SYQ C3H	GACCTTAGTGAGGACACCATTATTG
		CAGCTCTGCCATTGCCCATT
c103381.graph_c0	SYQ PAL	CATTGATCTGAGGCACTTGGAG
		CTCGCCATTGAAGCCGATTGTTA
c100482.graph_c0	SYQ HCT	GGTAAAGTACGACAGGCGATAG
		GAGCACCCAACAGTGTGGAAATT
c78409.graph_c0	SYQ HCT	GAGATCCAGCACCCAAAGAAC
		CTGACACCCACTCCTCCA

c100085.graph_c0	SYQ CSE	GACAACAAGATGGTCGGAAAGG<br>GCCGTGGATTCGACGCTAT

Table S2

Table S2. Sequence information of candidate genes

CCoAOMT<br><br>c102511.graph_c0	ATGGCCCAGAACCAGAGCCAAGAAGTTGGGAGGCACCAGGAGGTCGGCCACAAGAGCCTTTTGCAGAGTGATGCTC<br>TTTATCAGTATATACTCGATACCAGTGTGTACCCAAGAGAGCCGGAAAGCATGAAGGAGCTAAGAGATTTGACCTCCA<br>AGCATCCATGGAACATCATGACTACGTCTGCAGATGAAGGGCAGTTCTTGAACTTGCTTCTCAAGCTCATCAATGCCA<br>AGAACACCATGGAGATCGGCGTCTACACCGGCTACTCCCTTCTGGCCACCGCCCTTGCTCTCCCCGACGACGGAAAG<br>ATCCTGGCTATGGACATCAACAAAGAAAATTACGAGCTGGGTCTGCCGGTGATTCAGAAAGCAGGGGTTGCCCACAA<br>GATCGACTTCAGGGAGGGCCCTGCTTTGCCTGTTCTTGATCAGATGATCGAAGATGGGAAGTATCACGGGTCGTTCGA<br>CTTCATATTCGTGGACGCCGACAAGGACAACTATCTGAACTACCACAAGAGACTGATCGATTTGGTGAAGGTGGGAGG<br>ATTGATCGGCTACGACAACACCCTCTGGAACGGCTCGGTGGTGGCGCCACCCGATGCGCCGCTGCGGAAGTACGTCA<br>GGTACTACAGAGACTTCGTGTTGGAGCTCAACAAGGCTCTCGCTGCTGACCCTAGGATCGAGATCTGCATGCTTCCGG<br>TCGGCGACGGGATCACCCTCTGCCGTCGCCTTAGCTGA
C3H	ATGAACTCGAAGGGTGAATTGGACGAGCAGGGTTTGGAATTCAAGGCTATCGGGGCCGATGGGTCAAAGATCAGCGC<br>ATCAGTAACCGAGTACATGCCATGGCTTCGTTGGATGCTCCCCCTTGAGGATGAGGCGCTTGCCAAACTCAATGCCCG

c94054.graph_c0	TCGAAACCGACTCACTACAATTATCATGGAGGAACACACCCTTACCCGCCACATAACCGGTGGTGCCGAGCAGCATTT<br>TGTGGATGCTTTGCTCACCCTTCAAGATGAATATGACCTTAGTGAGGACACCATTATTGGGCTACTTTGGGATATGATTG<br>TTGCAGGAAGGGATACAACTTCCATTTCAGTTGAATGGGCAATGGCAGAGCTGATGAAGAACCCAAGGATCCAACTA<br>AAGGTTCAAGCTGAATTGGATCATGTAATCGGGGACCGGGTCATGACAGAATCTGATTTCCCCAACCTTCCTTACCTAC<br>AATGT
PAL<br><br>c103381.graph_c0	ATGGAAGCAATGAATCGTCAGGGAAGCAACAAGGTGGAGAGTTTCTGCGTGAGCGATCCACTGAACTGGGGAGCGG<br>CGGCGGAGACGTTGAAGGGGAGCCATTTGGATGAAGTGAAGCGCATGGTGGCGGAGTACCGGAAACCGGTGGTTCG<br>CCTCGGCGGTGAGACGCTTACGATATCCCAAGTGGCGGCTATCGCCCGGCGGGCGGAGGAGGTGAGTGTCGAGTTGT<br>CGGAAGCGGCAAGAGCCGGCGTGAAGGCCAGCAGTGACTGGGTTATGGACAGCATGAACAAGGGTACGGACAGCTA<br>TGGCGTCACCACCGGTTTCGGTGCAACCTCCCACAGAAGAACCAAACAAGGCGGAGCCCTTCAAAGGGAGCTTATCA<br>GGTTCTTGAATGCGGGAATCTTCGGAAATGGTACAGAATCTTGCCACACCCTGCCTCAGTCAGCAACAAGGGCGTCG<br>ATGCTGGTGAGGGTCAACACATTACTTCAAGGATACTCAGGCATCAGATTTGAGATATTGGAGGCCATCACCAAGCTC<br>ATCAACCACAACGTCACCCCATGCTTGCCTCTCCGGGGAACCATCACTGCCTCCGGCGACCTTGTTCCTCTCTCATACA<br>TAGCGGGGCTTCTGATCGGGAGGCCGAGCGCCAAGGCGGTGGGCCCCGACGGGAAGCCCCTCAATGCCGAGGAGGC<br>ATTCCATGTGGCTGGAATCAAGGGCGGGTTCTTTGAATTGCAGCCCAAGGAGGGGCTTGCTCTAGTCAACGGCACTGC<br>TGTTGGTTCTGCCATGGCCTCCATGGTTCTCTTTGAAGCTAACATCCTCGCAGTCCTGTCCGAAGTTCTGTCTGCACTT<br>TTTGCCGAAGTCATGCAAGGAAAACCCGAATTCACAGACCACCTCACTCACAAGTTGAAGCATCATCCGGGCCAAAT<br>TGAGGCTGCAGCTATTATGGAACACATTCTAGACGGAAGCTCTTACGTTAAAGCAGCTCAGAAGCTGCACGAGATGGA<br>TCCATTACAGAAACCTAAACAAGATCGATACGCCCTCCGAACATCTCCCCAATGGCTAGGCCCTCAGATTGAAGTTATC

	AGAGCAGCAACCAAGTCCATTGAACGGGAGATAAACTCCGTAAATGACAACCCCTTGATCGATGTTTCCAGGGATAAG GCTATTCATGGTGGTAATTTCCAGGGGACCCCAATCGGTGTTTCCATGGACAACACCAGGCTTGCCATTGCTGCTATTG GAAAGCTTATGTTTGCCCAATTCTCCGAGCTTGTTAATGACTTCTACAACAACGGGTTGCCTTCAAATCTGTCCGGTGG CCGGAACCCGAGCTTGGACTACGGCTTCAAAGGGGCCGAGATCGCGATGGCCTCTTACTGTTCGGAGCTGCAGTTTCT TGCTAATCCTGTCACCAACCATGTCCAGAGCGCTGAGCAGCACAACCAAGATGTGAACTCTTTAGGACTTATTTCTTC GAGAAAAACTGCTGAGGCTGTGGATATACTGAAGCTGATGTCATCCACTTTCTTGATTGCTTTATGCCAATCCATTGATC TGAGGCACTTGGAGGAGAATCTCAAGAACACAGTGAAGAACACTGTCTGCCAAGTGGCCAAGAGAGTGTTAACAAT CGGCTTCAATGGCGAGCTTCACCCTTCCCGGTTCTGCGAGAAGGACCTGCTCAAGATGGTCGACAGAGAACATGTTTT CGCATACATTGACGATCCCTGCAGCTCCACTTATCCATTGATGCAGAAGCTAAGGCAAGTCCTGGTCGAGCATGCCCTG AACAATGGAGAAAGGGAGAAGAACTCCAGCACTTCCATCTTCCAAAAGATCGTAGCCTTTGAGGATGAACTAAAAGC CCTTCTGCCCAAGGAAGTGGAGAGCACAAGAATCGACTACGAGAATGGAAATTCTGCCATTCCAAACAGAATCAACG ACTGCAGATCCTACCCTCTTTACAAATTTGTGAGGGAAGAACTGGGAGCAAGATTCCTAACGGGAGAGAAGACTACA TCCCCAGGTGAAGAATGTGACAAGGTCTTCACAGCAATGTGTGCTGGGAAGTTGATTGATCCGCTGCTGGATTGTCTG AAGGAGTGGAATGGAGCTCCTCTTCCCATCTGCTAG
HCT c100482.graph_c0	ATGGCGAAATTGATGGCAACCTACACAGTAAAACCAGCCAAGGAGACACCAGGAGGCTACAAATGTTTATCAGAGTG TGATCAAATCAAAGCTGTCACTCACGCCCCCACCATCTACTTTTACCCACCTGGAAATTTGTCGCTTGAGACGGTCGCC GACATTCTGAGAAACTCTTTGAGTGAGGCTTTGGCCATTTTCTATCCACTGGCCGGACGGCTGCACATTATCGATAGCG GCGGCCGCCTTAAGCTCGACTGCAATGCGGCGGGGGCTTTGCTCGTTGCAGTCGAATCGGAAGCTAAAATCGATGACT TTGGAGATTTCAGACCAACCCCGGAAATCCGAGCTCTCATCCCTCGCGTGGATTACAACAAGCCGATAGATGAACTGC

	CGTTGCTGGTGGTGCAGGTCACGAAATTCAGCTGCGGCGGACTGAGTCTGGGCTTGGGAATATCGCATATTCTCGTGGACGGCCTTTCTGCATTAGATTTCATCTCCGAGTGGGCTAAAATAGCCCGCGGCGAGGGCCCCGGCAGCCCCCCGCCGTTTCTTGATCGGTCCGTTTTCCGAGCGCCGGAGCATTTGACGGCGCCCGTGTTTGATCATCCAGATTTTGGTCCTCAGCCCCTCTTGATTGGGCAGAAAGATGACGTGGAAGAAAGAAAGAAAGAAACCTCAGTTCTCTTGTTGAAGTTAAGCAAAGTGCAGACCGAGAAATTGAAGGAAAAGGCCAACGAGGATGAAAACGTTTCCAATGGCCGGCGGCCGTACACAAGGTACGAGGCTGTTGCCGGACACATATGGAGGACAGCATGCAAGGCACGTCAGCATGAAGCCAATCAGCCGACATCGTTGTATATCGCCGTTGATGCTCGCAACCGCATGGAGCCGCCATTGCCGCCGAGGTATTTCGGAAATGCTGTTTTTCGGATAACTGCTGAAAGCACGTCAGGGGAGCTGGTGTCAAAGTCGTTAGGATATGCTTCTGGTAAAGTACGACAGGCGATAGAGAAGGCAACGCCTGAATATTTACAATCATGTCTTGTGTTTGTGAAAAATCAGAAAGATGTGACCAAGTTTAGGAATTTCCACACTGTTGGGTGCTCGAAAGGAGCATTTTATGGAAACCCTAGTATTGAGATCACTAGCTGGGCAAGGTTACCCGTCTATGGGGCGGATTTCGGATGGGGCCGGGAAATATACATGGGTCCTGGAGATTTAGGGTTTGATGGGAAGGTTTTCTTGATTCCAGAACAGGGTCTAGACGGATCGTTCTTTGTAGCATTGAGGCTGCAAGTACGCCACATTGATGCTTTCCACAAATTCATTTACCAAGACATTTAA
HCT c78409.graph_c0	ATGGTGCCCGAAGTGGAAGTGCACTCCAAGGAGACCGTAAAGCCATCTTCTCCCACTCCAGACCACCTCCGTCGATACCAGTTATCCTTTCTTGATCAAATATCTCCTCCAGTTTTCATGCCCATGACCTTCTTCTACCCGCCAGACCCTGATAGCAGATTCAGCCACACAGACATATCAAACAAGCTTAAGCAATCTCTCTCTGAGAATCTAGTCCGGTTCTACCCGTTAGCAGGTCGGGTTAAGGACGGTTTCTACGTCCTCTGCAACGATGAGGGTGTGCCCTACATTGAAGCCAGAGTAAAGGGCCAACTTCTGAGACGATCCGAGATCCAGCACCCAAAGAACTGCACAAATTCCATCCATATCAACTGGATGATGTCAAAGACTTGA

CSE<br><br>c100085.graph_c0	ATGTCGTCGGACTCTGGAATTTCGGCCAACTTCTGGGGCGATATGCCGGAGGAGGAGTACTATACCTCCCAAGGGGTG<br>CGCAACACCAAATCATACTTCGAAACCCCCCACGGCAACCTCTTCACCCAGAGTTTCCTCCCTTTGGATCTCCCCATCA<br>AGGCCTCCGTCTACATGAGCCACGGCTACGGTTCCGACACCGGCTGGCTCTTCCAGAAGATCTGCATCAACTTCGCCT<br>CCTGGGGCTACGCCGTCTTCCCCGCCGACCTCCTCGGCCACGGCCGCTCCGACGGCCTCCGATGCTACCTCGGCGACA<br>TGGAGAAGGTCGCCGCCGCCTCCCTCTCCTTCTTCAAGAGCGTCCGCACCAGCGAGCCCTACCGCCACCTCCCCGCCT<br>TCCTCTTCGGCGAGTCCATGGGCGGAGCCGCCACCATGCTCATGTACTTCCAATCGGATCCGGATCTCTGGACCGGCCT<br>GGTCTTCTCAGCCCCACTCTTCGTGATTCCCGAGAACATGAAGCCATCGAAGGCCAGGCTGTTCATGTACGGACTGTT<br>GTTCGGGATGGCTGACACGTGGGCGACGATGCCGGACAACAAGATGGTCGGAAAGGCGATCAAGGATCCGGAGAAG<br>CTGAAGGTGATAGCGTCGAATCCACGGCGGTATACTGGTCCGCCGAGGGTGGGAACGATGCGGGAGCTGCTTAGGGT<br>GACCCAGCACATACAGGATAATTTCTCGAAGGTAACGGCGCCGTTCTTGACGGTGCACGGGACGGCGGACGGGGTGA<br>CATGTCCGACATCGTCGGAGCTGCTGTACGAGAAGGCATCGAGTGAGGACAAGACATTGAAGATGTACGAGGGGATG<br>TATCATTCGTTGATACAGGGAGAGACTGACGAGAATGCAAATCTGGTGTTGAAGGATATGAGGGAGTGGATTGATGCG<br>AGGGTTGAGAGATATGGACCCTACAAATCCTAG
